# Good organizational practices to encourage women high-performance coaches in sports

**DOI:** 10.3389/fspor.2023.1287400

**Published:** 2023-11-27

**Authors:** Damien Taylor, Clare Hanlon, Andrew Dawson

**Affiliations:** Institute for Health and Sport, Victoria University, Melbourne, VIC, Australia

**Keywords:** good organizational practices, women high-performance coaches, senior managers, institutional work, organizational level

## Abstract

Women play a vital role in professional sport on and off the field. Globally, the dearth of women high-performance coaches in sport is a concern. For example, in Australia women represent 15% of high-performance coaches. One reason could be due to the lack of knowledge on good organizational practices that encourage women in this role and the overwhelming knowledge of practices focused on barriers for women high-performance coaches. The purpose of our research was to determine what good organizational practices exist to attract, develop, and retain women as high-performance coaches in Australia. Using a qualitative research design, semi-structured interviews were conducted with two study groups that comprised 16 women high-performance coaches and 13 senior managers from five National Sport Organizations (NSOs) in Australia. Data analysis was guided by practices that influenced the attraction, development, and retention of women high-performance coaches. Practically, findings revealed 12 good organizational practices and 31 associated recommendations to assist senior managers from NSOs in their quest to encourage women high-performance coaches in their sport. Theoretically, our research “reverses the lens” of the Ecological Intersectional Model (EIM) at the organizational level whereby the focus turns to good organizational practices rather than barriers for women high-performance coaches.

## 1. Introduction

Women high-performance coaches are recognized as role models and leaders within their sport and can inspire other women, men, girls and boys to become coaches (1, 2). Attracting, developing, and retaining women high-performance coaches however has been an ongoing problem. Literature that explores leadership roles in national sport organizations (NSOs), such as women high-performance coaches, highlights the dearth of women globally in these positions (3) and is reported to be between 6% and 14% (4–6). Subsequently, what emerged from the literature was despite several decades of research that examine the needs of female coaches, numbers continued to decline or stagnate (1, 7). In Australia, these statistics are no different, particularly for women high-performance coaches. At the Rio Olympics, the percentage of women high-performance coaches who represented Australia was 9%, and at the Tokyo Olympics it was 13%, currently it is less than 15% overall (8–10).

**Figure 1:**
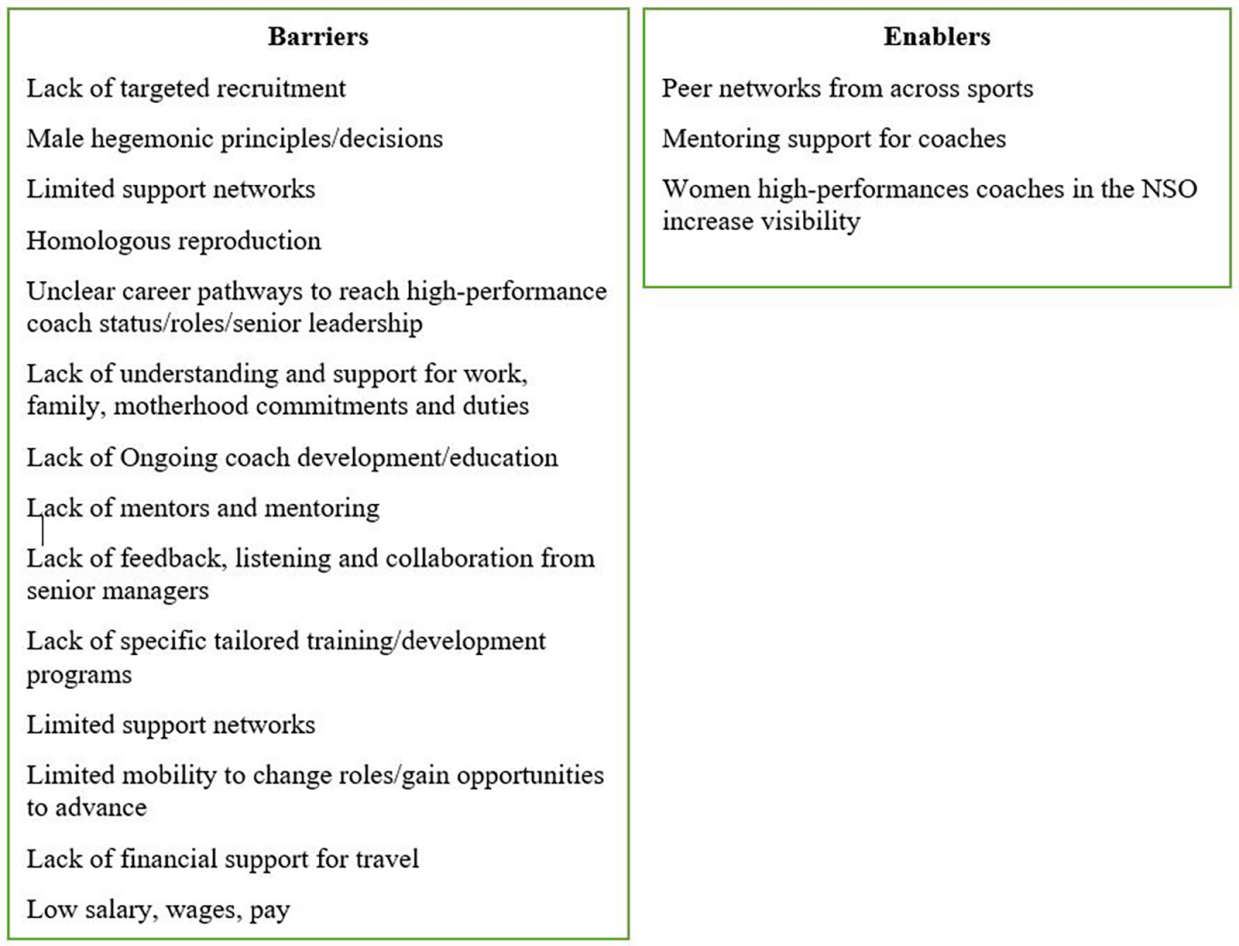
Organizational practices barrier and enablers.

Despite decades of research, minimal literature exists on organizational practices that encourage women high-performance coaches (1, 11, 12). Instead research has predominantly focused on organizational barriers for women high-performance coaches, primarily from the coach's perspective to the extent this knowledge has become well-known (7, 13). Moreover, past research revealed barriers and limited support practices exist within the organizational level (14–16). The depth of the problem is revealed in the list of barriers compared to enablers identified in Figure 1.

Researchers believed that to assist with the removal of the multitude of organizational barriers, a different approach and perspective is required (13, 15, 17). One that focuses on good practices that can attract, develop, and retain women high-performance coaches. These researchers believed that doing so would address the continued call for action, to delve into organizational practices that encourage women high-performance coaches (7, 13–15, 17, 18) and are implemented by senior staff (19).

### 1.1. Organizational practices barriers and enablers

Barriers that have deterred women to become high-performance coaches include: lack of targeted recruitment, male hegemonic principles/decisions (20), limited support networks (1), homologous reproduction to continually hire male high-performance coaches (4), unclear career pathways, and the lack of understanding and support for work, family, motherhood commitments and duties (21).

Continued challenges specific to senior managers include: the provision of ongoing coach development/education (17) and mentors and mentoring (15); enabling feedback, listening and collaboration and specific tailored training/development programs (2) and promoting support networks (22). The importance of mentors has been consistently recognized as a vital resource (7, 23) to support women high-performance coaches. In particular, mentors were highlighted for the support, knowledge, confidence and encouragement they provide for the coaches (14). Numerous economic barriers exist. These include: limited mobility to change roles/gain opportunities to advance (21, 24), lack of financial support for travel, limited support networks (7), low salary (25), and the lack of understanding and support for work, family, motherhood commitments and duties (17, 26). These barriers have been highlighted in the literature for more than a decade with the intention of encouraging policy makers and managers at NSOs to provide opportunities to advance roles, provide financial support for travel, review salary, and understand the significance of family, parenting, and work balance. Researchers have stressed that these barriers create inappropriate practices enacted by senior managers in sport (7, 14, 15).

Fewer enablers to encourage women high -performance coaching were identified. A key enabler was the visibility of women as high-performance coaches. Visibility where NSOs showcase these women on social media and celebrate the value and benefit these women provide as leaders, role models, and team members (7, 21, 27). Visibility provides evidence that women as high-performance coaches is achievable.

### 1.2. Organizational level of the ecological intersectional model

To adopt an enabling approach that moves beyond existing literature, we were guided by the Ecological Intersectional Model (EIM, 18) based on its focus on practices at the organizational level. The model comprises four levels that enables a broad-spectrum analysis of individual development. The analysis is conducted through interaction within multiple systems and environments including: individual/intrapersonal (e.g., personality, beliefs, values); interpersonal level (e.g., parents, friends, colleagues); organizational/structural level (e.g., organizational policies, organizational structures, professional practices); and social-cultural (e.g., gender stereotypes, cultural systems, and the effects of leadership). For the purpose of our study, the examination we focused on the organisational level, in particular, on the good practices perceived by women high-performance coaches and senior managers to attract, develop, and retain women in high-performance coach positions.

Organizations are complex, multi-layered entities with many tiers (28). Extensive research has examined organizations (institutions) to analyse their financial and economic performance, goals, strategies, and plans (29–31). The actors (people) within an organization contribute to achieving these performance measures, goals and strategies through the practices conducted, this is recognized as institutional work (32).

The institutional work of people shapes practices that create, maintain, or disrupt institutions (32, 33). The unique lens of our study examines the organizational level of the EIM through institutional work to address the purpose of our research, what good organizational practices exist to attract, develop, and retain women as high-performance coaches in Australia?

## 2. Method

To achieve the purpose of this exploratory study, qualitative research was deemed the most suitable. Our research is part of a larger study that comprised two qualitative phases. Phase one comprised documentary analysis of publicly available strategic plans from NSOs that met the 2019 Australian Institute of Sport (AIS) high-performance tiered funding criteria or were recognized as the top six independent NSOs in Australia. These plans needed to include targets for women high-performance coaches. Findings from Phase one identified five NSOs who recognized women high-performance coaches in their strategic plans and included Boxing Australia, Golf Australia, Rowing Australia, Swimming Australia, and Taekwondo Australia. These organizations were invited to be involved in Phase two of our study, which is the focus of this paper. This investigation focused on multiple case studies to understand the similarities and differences (34), between coach and manager perceptions on good practices, and to analyze data within each case and across the two cases (35). We acknowledge that just because these NSOs have recognized the need to focus on women as high-performance coaches in their strategic plans, it does not necessarily mean they are conducting good organizational practices. What it does mean is the managers in these NSOs are seeking or implementing good practices and our study will identify what these are and how they align with the perceived good practices from women high-performance coaches in their sport. Semi-structured interviews were conducted with women high-performance coaches and senior managers from the NSOs identified in study one. Senior managers comprised Chief Executive Officers (CEOs), directors of high-performance and high-performance managers. A benefit of these semi-structured interviews was the structure and flexibility that the method provides (36). A strength of multiple case study research is it allows for wider exploration of the research questions and theory building than single cases studies (37). A deductive and inductive analysis allowed for a comparison of narratives conveyed by the coaches' lived experiences from the implementation and management of organizational strategies to those of senior manager narratives who implemented the strategies (38).

### 2.1. Participants

Two case study groups were formed, recognized as study-one and study-two. Study-one comprised 16 women who fit the criteria: current high-performance coaches and coached at the Olympic Games, Commonwealth Games, or World Championship levels from 2016 onward. The reason for selecting 2016 was due to the public outcry, including the CEOs at the time of Sport Australia, Kate Palmer and the Australian Institute of Sport, Peter Conde, who noted that of the 160 registered high-performance coaches that represented Australia at the 2016 Rio Olympic Games and only 15 (9%) were women. The women's high-performance coach demographic characteristics comprised: Two participants that had more than 30 years' experience as a high-performance coach. Three had 20–30 years' experience and seven coaches had 10–20 years of experience. The remaining four coaches had less than 2 years' experience. The mean duration of coaching experience was 17 years (Table 1).

**Table 1:** Women high-performance coach participant demographics.

Participant alias	Years as high-performance coach	Number of sports as a high-performance coach	High-performance coaching experience at an international NSO
Aish	30	1	No
Samantha	22	2	Yes (2 NSOs)
Megan	15	1	No
Tayla	13	1	No
Cynthia	12	1	Yes (1 NSO)
Daisy	10	1	Yes (1 NSO)
Ebony	10	1	Yes (1 NSO)
Dawn	10	1	No
Matilda	7	1	No
Darcy	5	1	No
Candice	5	1	No
Serena	5	1	No
Florence	3	1	No
Evonne	1.5	1	No
Nadia	1	1	No
Steffi	0.2	1	No

Participants in study-two comprised 13 senior managers that included CEOs, high-performance managers, and/or managers of high-performance coaches. Most (*n* = 10) of the senior managers were male. Roles included: CEO (two male, one female), Chief Operating Officer (COO) (one female), high-performance directors (three male), high-performance pathway managers (four male), deputy performance director (one female), and director of coaching (one male). The combined average of experience as a senior manager was 15 years. The most senior management experience was 32 years and the least was 3 years. Only two senior managers had been in their current role for 10 years or more, with average employment in their current roles being 4.3 years. Two new senior managers, one with 18 months and one with 6-month experience, were women who left their coaching roles to become senior managers due to the opportunity this role provides compared to the constraints of coaching associated with being a mother and having family commitments (Table 2).

**Table 2:** Senior manager participant demographics.

Participant alias	Gender	Number of NSOs where they have worked as a senior manager	Senior management experience at an international NSO	Previous experience as a high-performance coach at an NSO
Steve	Male	8	Yes (2 NSOs)	No
Charlize	Female	5	No	No
Arnold	Male	4	No	No
Bruce	Male	4	Yes (1 NSO)	No
Sylvester	Male	2	No	No
Keanu	Male	2	No	No
Kyle	Male	2	No	No
Ryan	Male	1	No	No
Joel	Male	1	No	No
Trent	Male	1	No	No
Michael	Male	1	No	Yes (1 NSO)
Halle	Female	1	No	Yes (1 NSO)
Michele	Female	1	No	Yes (1 NSO)

### 2.2. Procedure

An invitation was sent to the CEOs from each of the five identified NSOs, to invite their organization to be involved in the research. The invitation noted the two groups of participants required for interviewing and the approximate 45-minute duration for each interview. Every CEO accepted the invitation to be involved. In doing so, the CEOs invited staff for each study cohort to voluntarily participate in the interviews. These CEOs appointed a manager to coordinate the project within their organization. The project manager, with the assistance of the researcher, invited participants to attend the interviews via email. The email contained project information and consent forms for participants to review and approve.

The interview questions focused on the organizational practices that each of the five NSOs used to attract, develop, and retain women as high-performance coaches. In total 13 questions (refer to Table 3) were posed to the two cohorts to allow for cross-check comparison analysis (39).

**Table 3:** Questions posed in semi-structured interviews.

Individual characteristics
1. How long have you been involved in sport as a manager?
2. How long have you been in your current management role?
Current state
3. What do you think attracts women to become a high-performance coach?
4. How do people in (organisation name) assist to attract and retain women as high-performance coaches?
5. What development has (organisation name) provided the women high-performance coaches?
Future state
6. What strategic practices by (organisation name) would attract women as high-performance coaches?
7. What strategic practices by (organisation name) would develop women as a high-performance coach?
8. What strategic practices by (organisation name) would retain women as high-performance coaches?
9. What support practices by (organisation name) do you believe the women high-performance coaches would like to have implemented?
10. Based on the (organisation name) strategic plan, explain any focus areas you think should target women high-performance coaches?
11. Based on the (organisation name) high-performance plan, explain any focus areas you think should target women high-performance coaches?
12. Explain how the overall sport environment affect the retention of women as high-performance coaches?
13. Are there any final comments you would like to make?

### 2.3. Data analysis

Data analysis consisted of three steps including: open, axial, and selective coding in order to generate a pattern of themes and create new theory from the data, rather than to begin with a theory (40, 41). Data collected from the semi-structured interviews, were prepared for deductive analysis according to the three central themes: attraction, development, and retention of women high-performance coaches (open coding). Each transcript was then analyzed for codes on the type of practices conducted within each theme (axial coding). Once analysis was conducted within each study group, results were merged, cross-checked and analyzed to explore similar and different responses (selective coding). Initial coding was cross-checked between the authors several times during the data analysis. The benefit of cross-checking is to determine whether the authors are in agreeance on the codes used and the coding (42). After this process the authors provided subsequent feedback to ensure a strong coding process was conducted, which added strength and validity to the process (39).

## 3. Results

Three types of good practices emerged from the data focused on attraction, development, and retention of women high-performance coaches. Primarily, participants identified good practices as supportive practices. Inductive themes also emerged including the institutional work of actors, in particular, senior managers. Similar findings were evident between the two groups however differences also existed.

### 3.1. Attraction practices

Five codes evolved as good practices to attract women high-performance coaches (refer to Table 4). Three codes were recognized by over half of the participants and included the need to: recognize flexibility of family needs, talent identification, and transparent career pathways. To a lesser extent, the two remaining practices included opportunities to lead in training camps and showcasing women high-performance coaches. While the two participant groups identified similar practices, perceptions also differed between the two cohorts including pathways for attraction and talent identification.

**Table 4:** Participant responses—attraction theme.

Good organisational practices	Women HP coaches participants (*n* = 16)	Senior managers participants (*n* = 13)
Flexibility with family needs	13	9
Talent identification	11	11
Transparent career pathways	10	13
Training camps	7	5
Showcase women high-performance coaches	6	6

#### 3.1.1. Flexibility with family needs

The need to recognize and understand the associated pressures and family responsibilities for women high-performance coaches was clearly articulated by participants. The need to schedule time for family responsibilities was noted in particular by coaches (81%) who specifically appreciated senior managers trying to manage time and schedules to fulfil their parent and high-performance coach roles. In doing so the visibility to potential new coaches of the flexible family-focused environment was created that eliminated the difficulty of forcing women high-performance coaches to make a choice between motherhood or their career:

As soon as you start the family, that is where the problem starts. That's why female coaches stop coaching because they have to either look after the kids or they have to pay for it (Cynthia, coach).

To help attract women to high-performance coaching roles, most senior managers (69%) also acknowledged flexible schedules to meet family commitments and fulfil high-performance coaching responsibilities. Flexibility requires a collaborative approach between women high-performance coach candidates and direct senior managers. Arnold (senior manager) suggested that:

I think there are elements of that where you need to get to a point where it can become a proper two-way discussion…on what the choices are, and how do we make that work… Because it's only ever seen as a reason why you can't, not as a way of how do we make it work?

#### 3.1.2. Talent identification

Talent identification was noted by senior managers (85%). Both participant cohorts noted that identifying women high-performance athletes on the cusp of a career transition with the potential skills required to coach was good practice. In this case, NSOs appointed dedicated senior managers to identify women athletes as potential coaches and ensure a smooth transition into national coaching programs. Structured talent identification parameters established by senior managers ensure correct processes and criteria are applied. Ryan (senior manager) noted a talent ID framework was effective to identify and attract talented women to become high-performance coaches stating “We run the programs within each of our states, in terms of how we set that up, and why we need to apply similar sorts of principles to identifying coaches”.

Additional talent identification practices recognized included senior managers enabling opportunities for women to become high-performance coaches, creating transparently structured coach processes and management, and establishing a talent pool of women high-performance coaches. Joel (senior manager) created talent identification opportunities similar to athletes:

Providing talent identification opportunities [is important], just like an athlete. If you've got a young athlete, you provide tournament opportunities, you provide coaching opportunities. If you're looking for a person to fulfil a role, I think along the journey, you provide opportunities for them to see if they are actually the right fit or see if they actually have the love and passion for that role… It goes back to the more females that are playing your sport, you have more talent to choose from to be your coaches.

To be approached as a potential coach was an important practice recognized by coaches. These participants (69%) articulated that the “tap on the shoulder” was a pivotal factor that encouraged them to apply for their high-performance coach role. An element of self-doubt by these women on the required skills and knowledge when applying for high-performance coach roles was articulated by the majority of coaches (69%). Self-doubt related to their perceived lack of required skills and experience to apply for a role and was one reason these women believed a low number of women in these coach positions existed. These participants articulated that the “tap on the shoulder” was a pivotal factor that encouraged them to apply for the high-performance coach role, as noted by Aish (coach):

There was that tap on the shoulder, would you be interested? And then, I went, well I'll go and put my hat in the ring and then got the job. And that's how I started. And then, once I did that, there was this realization of I'm not going to be a world champion athlete, but maybe I can be a world champion coach.

With the encouragement from senior managers self-doubt was eased and confidence raised to apply for these positions.

#### 3.1.3. Transparent career pathway

Defined and clear pathways for a career in high-performance coaching was noted as a good practice by both cohorts. All senior managers recognized transparent career pathways were important to support women in high-performance coaching roles. Several good practices to support transparency were indicated by senior managers as a necessity to formalise and incorporate. Specifically, defined clear pathways for (1) a high-performance athlete to become a high-performance coach, and (2) for non-athletes who aspire to become a high-performance coach. Additional good practices recognized by managers include the provision of legitimate opportunities, support women, and establish a target percentage to increase the number of women to become high-performance coaches.

The majority (69%) of coaches explained what entailed defined and clear pathways. Clarity on expected experience, demonstrated skills, and formal qualifications were identified. Pathway clarity was continually repeated by coaches, as noted by Dawn (coach), “I think it's really important to identify what the pathway looks like for a high-performance coach. What does a high-performance coach look like? I don't think that's even been established”.

#### 3.1.4. Training camps

Opportunities to attend and lead at training camps were recognized as good attraction practices. Opportunities within these camps include the experience of being surrounded in an intense high-performance culture, environment, standards, and stakeholder expectations. These camps provided proactive and real-time opportunities to gain new knowledge and advance skills on strength and conditioning, medical knowledge, leadership, and management under high pressure and expectations to deliver results. The investment to actively involve women high-performance coaches at international training camps was highlighted by Ryan (senior manager) “actually investing in them and showing the system that you're going to develop them to be a great coach that's going to have these experiences”.

Several notable differences were evident between the coach and senior manager perspectives on enabling opportunities within these training camps for coaches. In particular, coaches focused on the provision of opportunities to not only attend but lead at international training camps and competitions, whereas managers referred to administrative task opportunities that had little to do with leading athletes and other coaches at these camps. To know opportunities existed to attend international training camps and be part of the leadership team to execute their knowledge and skills, was highly regarded as an attraction practice by the coaches. Whereas to experience high-pressured international coach environments and gain new learnings to improve their own coaching skills, leadership and management practices were recognized by senior managers.

#### 3.1.5. Showcase women as high-performance coaches

To showcase women high-performance coaches was recognized as a good attraction practice by coaches and senior managers. In particular the recognition of outcomes and impact they create in the coach role. To celebrate and increase awareness and visibility of these successful coaches resulted in them becoming role models that assisted to attract future generations of women in coaching. The visibility of women high-performance coaches as role models attracted Cynthia (coach) to coaching beginning in her youth:

Where I grew up, we would watch Olympics on TV. We would watch every single sport. We had lots of female coaches and what they said, how they said it and how they deal with this and that. It was so interesting.

### 3.2. Development practices

Three codes evolved as good practices to develop women high-performance coaches (refer to Table 5). These included the provision of: ongoing development of high-performance coaching skills, mentors, and a safe working environment. Both participant cohorts referenced each code similarly, however, different cohort perspectives existed on what constitutes a safe environment.

**Table 5:** Participants responses—development theme.

Good organisational practices	Women HP coaches participants (*n* = 16)	Senior managers participants (*n* = 13)
High-performance coach skills	14	10
Provision of mentors	8	5
Enable a safe working environment	6	6

#### 3.2.1. High-Performance coach skills

The provision of support to enable ongoing improvement to increase knowledge, skills, and experience of coaches was deemed important by the majority of coaches (88%) and senior managers (77%). In particular specific skills related to leadership, management, coaching, and communication.

Tailored personal and skill development from an individual perspective was recognized as good practice by these coaches. Senior managers played a key role to enable this development, through collaborating with each woman high-performance coach in their sport to provide proactive feedback and assist with self-assessment and ongoing improvement. Serena (coach) relayed her enthusiasm for personal development and feedback to continually improve:

I love feedback. I love learning and I love developing. That's why, I suppose, I became a high-performance coach. I think a lot more development. Personal development is not only sport-specific skills.

#### 3.2.2. Mentors

The provision of mentors to enable additional support, guidance, and feedback was acknowledged as a key development good practice. The role of a high-performance coach was recognized by participants to change and evolve, thus the need for mentors to assist them through this process. Mentors enabled personal and professional development including the provision of guidance, feedback, and reassurance. Samantha (coach), who has coached for 40 years, recognized the influence of her mentor and in return her focus to mentor other women high-performance coaches: “It's such a powerful thing, the mentor training. It's really, really powerful. I see the results, all the time. It's also very developmental for the mentor as well”.

These mentors tend to be experienced high-performance coaches, potentially from different sports, and have experienced a range of situations. Participants recognized mentors provide an influential and supportive platform to listen to challenges that arise and through their experiences help navigate pathways and outcomes to assist the coaches.

#### 3.2.3. Safe working environment

Despite the recognition of safe workplace environments as a good practice to help develop women high-performance coaches, the definition of these environments varied between the senior manager and coach cohorts. Senior managers referred to specific situations as safe environments whereas the coaches described these environments from a broader perspective.

Senior managers recognized the importance of enabling opportunities for women coaches to speak openly and freely without fear of ridicule. Coach development programs was one example provided that allowed a safe environment for these coaches to make mistakes and learn through the expertise of others. Trent (senior manager) summed this up as:

Creating an environment where it's okay to speak up and have your voice and not feel like you're going to get ridiculed, and you can make mistakes. I think that's a really important aspect of growing as a coach.

The broad perspective noted by coaches included the provision of a safe environment at courses, programs, training camps, or seminars to reduce apprehension, encourage engagement, and show vulnerability when actively participating. The importance of a safe environment was noted by Steffi (coach) as one that provides an opportunity “where I feel like I can still grow and really develop and contribute”.

### 3.3. Retention practices

Four codes evolved for good practices to retain women high-performance coaches (refer to Table 6). Three were recognized similarly by both cohorts: regular communication, peer networks, and advocacy for women as high-performance coaches. Motherhood and parenting support differed in perspective and was referenced by most coaches (81%) for good practice and only half of the senior managers.

**Table 6:** Participant responses—retention theme.

Good organisational practices	Women HP coaches (*n* = 16)	Senior managers (*n* = 13)
Regular communication	13	9
Peer networks from across sports	13	9
Motherhood and parenting support	13	7
Advocacy for women high-performance coaches	12	13

#### 3.3.1. Regular communication

Regular communication between senior managers and women high-performance coaches was prominently acknowledged by coaches (81%) and to a lesser extent by senior manager (69%) respondents. One-on-one communication provided opportunities to voice concerns from a woman-coach perspectives, enable feedback, and feel listened to and supported. Weekly one-on-one communication between senior managers and these coaches was deemed a good practice by the coach cohort. As typified by Serena (coach): “[My manager] being there weekly. Being a lot more present in the current environment. To be the support and to be there”.

One-on-one communication with these women coaches was articulated by senior managers as an opportunity to listen to the coaches' perspectives for concerns, gain feedback on experiences, and create affirmative actions to support them. In doing so identify barriers, areas of concern, and establish strategies to enable these coaches to rectify related issues and move forward, as highlighted by Arnold (senior manager):

Really talk to the female coaches and say …What are the things that are holding you back? What are the barriers? What are the ones that we need to acknowledge, what are the ones that [we] really have no control over and where do we get the biggest impact in changing strategy or addressing something?

#### 3.3.2. Peer networks

To encourage women high-performance coaches to engage in peer networks from across professional sports was deemed a good retention practice by coaches (81%) and senior managers (69%). Due to the lack of women high-performance coaches within NSOs, these peer networks were recognized to assist build their confidence, share information and decrease the sense of isolation. Peer networks were described as a positive, proactive space to discuss challenges, issues, share ideas, and provide support structures. As highlighted by Trent (senior manager):

I think utilizing other sports, given the low numbers, there's female coaches in AFL and cricket, hockey and water polo… is like engaging the coaches in something that I think can help as well. Some cross-pollination with coaches, and just some general guidance of “this is what we do to get here, and we'd suggest to do this and that”.

#### 3.3.3. Advocacy for women as high-performance coaches

Advocacy was acknowledged from several perspectives. The coaches recognized managers' efforts to promote, celebrate, and increase visibility of successful women high-performance coaches within their NSO as a good retention practice. Acknowledgement was gained that NSOs who advocated for women high-performance coaches helped grow awareness, support, and magnified their importance. As recognized by Steffi (coach):

I think the biggest strategic practices, is what they're currently doing now to be honest. [We should] continue this culture change of support and acknowledgement of valuing women and their potential to make significant contributions to our sport across the entire management [team]. Strategically show that to people in the community so that they can see there's promotion of women as a high-performance coach. Advocate for women in high-performance coaching roles, but across the entire management.

Similarly, senior managers recognized the need to advocate for women high-performance coaches. Advocacy that highlights the essential role these women play as a respected member of the high-performance coaching team. Advocacy included the need to “live” the advocacy through action and being an allied “voice” to stakeholders. In doing so assisted to develop a culture that supported practices for women as high-performance coaches.

#### 3.3.4. Motherhood and parenting support

Activation of supportive practices for motherhood and parenting were recognized by more coaches (81%) than the senior managers (59%) as a good practice to retain women as high-performance coaches. The need to understand and enable this support by NSOs was noted by Daisy (coach): “If we want to actually provide a career path for women within coaching, we have to have flexible conditions so that they can work around their family lives as well”.

Steffi (coach) reinforced the need for NSOs to implement practices that support and acknowledge motherhood and family roles to retain women high-performance coaches:

With a lot of women sometimes it is quite hard for them to stay because, they've got their families, other priorities as well. New mothers especially. I think again making sure that there are sustainable practices or things put in place for women to be retained.

Practices to support travel, start a family, and create family-friendly workplaces were articulated by both respondent cohorts. Flexible schedules to allow for parental responsibilities and workplace spaces to accommodate children or child minding were specific examples and recognized by Keanu (senior manager) “I think that's something that is more attractive, especially if they've got family and kids”.

Senior managers highlighted educating stakeholders on the importance of workplace spaces to accommodate children or child minding were good practice to retain women high-performance coaches. Charlize (senior manager) explained that through their education program to staff, a shift has slowly evolved at her NSO to understand and support motherhood and family friendly environments:

I've seen some shifts in the last couple of years that we're starting to be a lot more clear about women and children being in the environment [and] how we can facilitate that.

## 4. Discussion

Globally, a dearth of women high-performance coaches exists. In Australia for example women represent 15% of high-performance coaches (10). Barriers for women high-performance coaches are well-known (13, 15). What is not commonly known are organizational practices to encourage women in these roles. Good organizational practices to encourage women as senior leaders including high-performance coaches, are highly sought (14, 15).

The purpose of our research was to identify good organizational practices to attract, develop, and retain women high-performance coaches. These practices will help educate senior managers about facilitators that encourage women to apply for and remain high-performance coaches (43, 44). Findings from our research also addressed the limited good practices known to exist at the organizational level of the EIM (14–16). In order for these good practices to occur institutional work plays an important role. Institutional work recognizes people as actors whose actions shape practices that create, maintain, or disrupt institutions (32, 33). Actors such as senior managers in NSOs play a significant role in the outcome of these practices (45). Findings from our research reinforce the vital role senior managers play to enable good organizational practices to encourage women high-performance coaches.

Time spent as a senior manager at an organization and the number of organizations a senior manager has experienced can impact their practices, leadership and management that when working with stakeholders such as high performance coaches (46). These findings are supported by our research, specifically in high-performance sport settings such as NSOs. The senior managers in our study tended to have worked for multiple NSOs in Australia and abroad or had transitioned from coaching to management roles, giving them broad experience. As a result, these managers were receptive to adopting new practices that developed opportunities to attract, develop, and retain women high-performance coaches including the need to create family-friendly coach environments where children are welcome. These findings reveal that to embrace and promote leadership diversification in coaching related to women high-performance coaches, it is recommended that NSOs appoint senior managers who have cross-sport experience or who have transitioned from high-performance coaching to management.

Previous research has failed to shed light on organizational practices established by senior managers to attract, develop and retain women high-performance coaches, in consequence this topic has remained poorly identified and understood (7, 14, 15). Our research has sought to address this gap and identified 12 good organizational practices with 31 associated recommendations to encourage women high-performance coaches (Table 7). To dissect these practices in detail, the following section is presented in three subsections: attraction, development, and retention. Furthermore, each subsection provides associated recommendations to guide senior managers for good practice outcomes. A holistic approach to the EIM can now be developed that fills the gap at the organizational level to encourage women as high-performance coaches (14, 15).

**Table 7:** Good practices to encourage women high-performance coaches.

	Practice	Associated recommendations
Attraction	Flexibility with family needs	(1) Roster training schedules to non-school hours (e.g., between 10 am and 2 pm) (2) Establish on-site childcare (3) Roster days off for coaches on weekdays throughout the year
	Talent identification	(1) Identify recently retired women athletes with an interest to continue in the sport and approach them to become a high-performance coach. Encourage the skill set they have to become a high-performance coach and provide the support structures to attract them to the role.
	Transparent career pathways	(1) Provide a clear transparent selection criteria (2) Showcase a clear statement on skills and experience required as a high-performance coach (3) Promote internal and external scholarship opportunities that include provision for childcare and family responsibilities (4) Strongly encourage women to apply in the position description
	Training camps	(1) Senior managers to be involved in the planning process of coach roles during training camps (2) Senior manager to attend training camp (3) Women high-performance coaches to play an active leadership role in training camps (4) Enable women to coach male teams and athletes
	Showcase women high-performance coaches	(1) Understand the effects of not showcasing women high-performance coaches (2) Ask women high-performance coaches in their sport how they would like to be showcased to stakeholders to promote their strong skills set
Development	High-performance coach skills	(1) Establish a structured development process that includes a minimum of two collaborative meetings with women high-performance coaches to discuss knowledge and experience gaps (2) Tailor individual development programs (3) Enable attendance at leadership and management programs led by the AIS and any other agreed professional development providers
	Provision of mentors	(1) Identify suitable mentors for women high-performance coaches
	Enable a safe working environment	(1) Establish training camp structures that comprise high-performance coaches with strong skill sets in which women are highly represented (2) Activate a code of conduct signed by senior managers and high-performance coaches to provide welcoming and inclusive elite athlete training program environment for all coaches
Retention	Regular communication	(1) Establish open, clear, regular (preferably weekly) one-on-one communication (2) Listen and respond to needs communicated by the women high-performance coaches to help reduce ambiguity and frustration
	Peer networks from across sports	(1) Identify cross-sport coach professional development programs for women coaches to attend (2) Create online peer-coach networking opportunities sessions, e.g., Facebook groups, WhatsApp groups, online coffee catch ups (3) Encourage experienced women high-performance coaches to lead peer groups to assist with growth and development of them
	Advocacy for women high-performance coaches	(1) Be a strong advocate for women high-performance coaches (2) Call out inappropriate situations that may be faced by women high-performance coaches (3) Track the equitable acknowledgement of high-performance coach achievement for women and men
	Motherhood and parenting support	(1) Professional development for senior managers on the complexity of motherhood and parenting needs for coaches (2) Ask women high-performance coaches on their parenting needs (3) Enable family travel to competitions and training camps

### 4.1. Good practices to attract women high-performance coaches

Organizational practices to attract women become high-performance coaches has been ambiguous and problematic (12, 43, 47). As a consequence, barriers for women in this role are constantly recognized including: lack of understanding for work, family, motherhood commitments and duties (21), lack of recruitment, unclear pathways (16), male hegemonic principles (20), managerial hegemonic hiring decisions (4), and homologous reproduction (48). To reverse the barrier lens and attract women high-performance coaches, five good organizational practices and 14 associated recommendations were commonly recognized by women coaches and senior managers in our study. These practices include: flexibility with family needs, talent identification, transparent career pathways, training camps, and showcase women high-performance coaches.

Flexibility with family needs requires understanding and support by senior managers. A key challenge is the need to juggle time between the demands of a high-performance coach and family responsibilities. The juggle has been previously identified (1, 49) however in the case of our study, practices do not seem to have changed. Such flexibility, has been problematic in the past. Potential conflicts with the demands of time required for women high-performance coaches in regards to their family responsibilities and job demand has been a long-term frustration and hindrance to attract women in the profession (1, 49). To enhance flexibility in the workplace, coaches in the current study highlighted the importance of allowing school drop-off and pick-up as a parent and when not provided anxiety resulted that conflicted with their role as a coach. Another example is flexible work schedules that enabled these coaches not to work on weekends. Senior managers in the current research who understood this conflict were aware that practices to support family needs required flexibility to accommodate these coaches. Despite this awareness, research continues to show good organizational practices, in particular related to flexibility in the workplace, are yet to be recognized (1, 7, 11, 26, 49, 50). Conscious and concerted action by senior managers needs to be publicly showcased that demonstrates a high-performance coach role can be flexible according to family needs. An example recognized in our study was enabling these coaches to conduct school drop-off and pick-up for their children.

Talent identification for women to become high-performance coaches requires proactive practices by senior managers. A lack of foresight or knowledge exists as an athlete on the ability to transition to a high-performance coach, however many athletes are passionate about remaining in their sport (51). Women high-performance athletes who became high-performance coaches in our study noted that without being approached by a senior manager on the coaching opportunity, a coaching career would not have been considered. To be approached enables an opportunity for former or retiring high-performance women athletes to pursue high-performance coaching roles.

Transparent career pathways for women to become high-performance coaches builds clarity and understanding on requirements and accessibility of these roles. A lack of transparency on set selection criteria, knowledge, skills, and experience needed for the role has been identified (27, 52). Our research identified inconsistent career pathways exist. In some cases, luck had secured women as high-performance coaching roles. These coaches noted the importance of a clear selection criteria that underpinned the skills and experience required for the role to assist attract women with strong skill sets to apply. A clear and transparent pathway criterion was identified in our study as an attraction source to encourage women to become high-performance coaches. Senior managers who provide clear pathway guidelines that include a clear statement on skills and experience required as a high-performance coach was a popular example.

The opportunity to lead at local through to international training camps was strongly recognized as an attraction to coach by women high-performance coaches in our study. Training camp opportunities have been previously recognized (11, 53), however two components evolved from our study that expands literature. The opportunity to actively coach at international training camps and to be a lead coach at these camps. In doing so helps create a gender-diverse high-performance leadership culture. In these situations women high-performance coaches were exposed to managing athletes and teams, meeting NSO and stakeholder expectations and experience how other countries differ in their high-performance programs when under pressure to perform at the highest level. Training camps were difficult to replicate at professional development workshops, instead the opportunity to lead at training camps was a key attraction factor.

To showcase women high-performance coaches internally and externally to the organization was warmly recognized by participants in our study. The absence of women visible in these roles can lead to their lack of confidence and self-belief (7, 16, 20). The battle to showcase women high-performance coaches has been recognized (17, 54), however, in the case of our study, senior managers recognized the positive effect celebration of these coaches provides to their sport and wider community. Recognition included senior managers: showcasing women in these roles as role models; encouraging media to interview these women; and introducing these women to external stakeholders and broader community, noting their skills as a high-performance coach rather than emphasizing their gender. The lack of visibility of women high-performance coaches is not a new finding, men are predominantly showcased in high-performance coaching roles (54–56). The consequence is this lack of visibility/showcasing makes it difficult for women to believe that being a high-performance coach is valued and achievable. Therefore, our findings reveal that showcasing the success of women high-performance coaches builds prominence in their sport for women as leaders. Not all women may feel comfortable on how they are showcased by their sport, in this case our study identified the importance of asking women high-performance coaches how they would like to be showcased to stakeholders on their strong skills.

### 4.2. Good practices to develop women high-performance coaches

High-performance coaches require diverse skills to lead and manage their athletes, teams, stakeholder's and organization's expectations (57). Ongoing coach development is required. Such development assists to build the quality of high-performance coaches including their leadership, management, psychological, teaching, social and cultural awareness, and mentoring skills (58–61). Ongoing coach development for women however is lacking in education (61), support networks (2), mentors and mentoring (22), specific tailored training and development programs (15), support from senior managers (16), and poor investment from senior managers (62). To develop women high-performance coaches, three good organizational practices and six associated recommendations were commonly recognized in our study including: high-performance coach skills, the provision of mentors, and enable a safe working environment.

High-performance coach skills require ongoing improvement. A challenge for women in these roles has been the opportunity to advance their knowledge and skills (20, 63). Women coaches have expressed the need to attend tailored development programs that include a focus for ongoing development and individualized rather than generic development programs regardless of the individual needs of each coach (64). Our study however, does not indicate these needs have been met. Instead our study identified the importance of co-designed programs for women high-performance coaches, co-created by senior managers and coaches within the sport. The opportunity then exists for these programs to be monitored based on how they cater for the individual needs of these women.

Unlike previous research that noted the need to provide mentoring skills to women high-performance coaches (22), the good practices identified in our study moved from the focus to “fix” women and instead focus on “fixing” organizational practices to encourage women into these coach roles. One example is for senior managers to identify relevant mentors to assist women in their coaching role, rather than women themselves trying to find mentors. Mentors help develop, support and provide valuable lived experiences to guide women high-performance coaches. Mentors develop coaching philosophies, knowledge, training skills, communication skills, and organizational relationships (11, 14). The responsibility for senior managers to identify and provide suitable mentors for women high-performance coaches reinforces the commitment the sport has to develop women in these roles.

The provision of safe workplace environments to assist the development of women high-performance coaches, is a good practice identified in our study and is yet to be explored in detail. Male-dominated sport environments negatively impact the development of women high-performance coaches (65–67). The ability to engage with stakeholders in programs, training camps or events without fear of ridicule or scorn was recognized in our study as aspects associated with a safe workplace environment. In doing so, women feel comfortable to openly and freely participate as leaders. Training camps is one example provided in our study on how a safe workplace environment is created, comprising women and men equitably represented as high-performance coaches. The environmental structures and practices at training camps were noted by senior managers as an area for review to be less daunting for women high-performance coaches. The provision of safe environments for the women high-performance coaches to benefit from the camps was to ensure the camp structure was open to women high-performance coach participation and respectful of all stakeholders. The coaches' broader perspective noted the environment experienced at courses, programs, training camps, or seminars can negatively or positively affect the learning outcome. In particular, coaches described safe environments that allow engagement and to show vulnerability without fear when actively participating to generate participants full attention in the program without apprehension. As such, the coaches highlighted these safe environments helped maximise development opportunities. Two examples provided by the senior managers to assist with safe environment creation were:

(1) Establish training camp structures that comprise high-performance coaches with strong skill sets in which women are highly represented
(2) Activate a code of conduct signed by senior managers and high-performance coaches to provide welcoming and inclusive elite athlete training program environment for all coaches

### 4.3. Good practices to retain women high-performance coaches

Organizational practices to retain women high-performance coaches has proven to be difficult (1). Notable barriers for coaches impacted their decision to leave their role rather than establish a long-term career as a high-performance coach (7, 63, 68). These barriers include: a lack of communication (69), support (17), and understanding for women high-performance coaches’ needs (18, 21, 70). To retain women high-performance coaches, four good organizational practices and 11 associated recommendations were commonly recognized by participants in our study. These practices include: regular communication, peer networks from across sports, motherhood and parenting support, and advocacy for women high-performance coaches.

Regular communication by senior managers to women high-performance coaches requires support from senior managers. A key challenge is the need to reduce ambiguity and lack of information sharing with women high-performance coaches. The desire for consistent, regular communication and information sharing to reduce uncertainty, feelings of isolation, and ambiguity for the coaches has been previously recognized (18, 69, 71, 72). Our study identified that regular information sharing and communication requires commitment from senior managers to regularly communicate with the women coaches. Good practices that typified regular communication were those instigated by senior managers to establish open, clear, regular (preferably weekly) one-on-one communication.

Peer networks from across sports provide essential support for women high-performance coaches. A challenge for women high-performance coaches are support networks and access to peers who can provide relief from the stressors of high-performance coaching. Peer networks from across sports have been recommended to assist with support and the retention of coaches (51, 64, 63). When senior managers develop these peer networks, our study identified that self-confidence increases for women high-performance coaches, active information sharing occurs, and resilience is built to remain a high-performance coach. In particular, findings identified two type of peer networks were important. First were internal peer networks, regardless of gender, established by NSOs for high-performance coaches was beneficial and enabled the move away from the reliance on these women to create their own peer networks. Second, were external peer networks targeted to women from across sports, to provide positive reinforcement, offer support to share experiences and ideas, listen to storytelling to help grow knowledge, and showcase experienced coaches as role models for new inexperienced coaches. Senior managers who enabled peer networking opportunities in the form of Facebook and WhatsApp groups, and online coffee catch ups, were identified examples of good practices in our study.

Advocacy for women high-performance coaches is important. The prominence, recognition and celebration of women high-performance coach achievements internal and external to the sport needs to be evident (68, 73). Genuine advocacy conducted by senior managers was recognized in our study to raise the prominence and highlight organizational appreciation of women high-performance coaches as leaders and role models. Such appreciation assists to retain these women. To track the acknowledgement of high-performance coaches regardless of gender could be a good retention practice activated by senior managers to ensure equitable advocacy is evident.

Motherhood and family support is a key challenge for women high-performance coaches. In particular the lack of practices and coach structures that enable support and balance for work and family. Historically, motherhood and family responsibility have been recognized as a stressor and continual barrier for women high-performance coaches to remain in the role (24–26). Our study identified that organizational structures and practices can be modified to enable the conduct of family responsibilities. The concern is however that despite the need from coaches for NSOs to recognise and support family needs, it was less recognized by senior managers as a good practice. In this case, to activate good practices related to motherhood and family support, senior managers need to be educated on the complexity of motherhood and parenting needs associated with being a high-performance coach. To assist form these supportive practices and structures, senior managers acknowledged the more informed they are on the complexity of motherhood and parenting needs for coaches, the more aligned they are to provide respective practices.

A focus over several decades is evident on the barriers for women to become high-performance coaches. A new perspective is provided from our research focused on organizational facilitators to attract, develop and retain women as high-performance coaches. Specifically, the identification of good practices enacted by senior managers to encourage women in these roles.

Our research explored good organizational practices recognized by women high-performance coaches and senior managers across five NSOs in Australia that helped attract, develop, and retain women in these coach roles. We acknowledge there are limitations in this research. Findings were gained from an in-depth analysis of 29 participants who were key stakeholders in the attraction, development and retention of women high-performance coaches. Quantitative data however would numerically validate the four factors and organizational practices identified in our study. There is an opportunity to explore if these practices have global relevance. We encourage global studies in this field, in order to learn from a diversity perspective on global good practices to encourage women as high-performance coaches. A further limitation relates to sampling where 10 of the 13 senior managers were men, responses on what good practices entail could potentially differ if the sample was equally represented by women or if the sample was dominated by women. This could be a note for future research. Another limitation is our research and the associated references are primarily western-oriented. We invite researchers to form a team of western and non-western representative for future studies to collaborate and thereby increase the contributions in richness and diversity. We encourage future research to explore a broader perspective with a global reach for studies in this field, as much as it should be important for researchers to also learn from a global diversity of investigations. The current references are “anglo-authors” centered. In future research, we invite authors to open the search focused on a more global perspective, increasing the contributions in richness and diversity.

The good practices identified in our study could become a benchmark for future research. Although ideally, it would have been beneficial to have validated with participants from our study, the recommendations that evolved, instead this could be an opportunity to translate these recommended practices into a framework that could be co-created with women high-performance coaches and senior managers to then pilot across sport to identify its effectiveness and fine-tune for potential roll-out across national and/or state sport organizations in Australia or more broadly. Another opportunity includes comparing the good practices gained in our study on women to those required by men high-performance coaches. In doing so would identify common and unique practices required for each gender.

## 5. Conclusion

Good organizational practices that encourage the attraction, development and retention of women high-performance coaches are not commonly identified, instead barriers are more prominently reported (1, 15). In consequence a plethora of barriers are evident at the EIM organizational level (14–16). The purpose of our study was to shift this focus to identifying good organizational practices that attract, develop, and retain women high-performance coaches. In doing so, our study has advanced knowledge that enables senior managers to encourage women high-performance coaches. The results of our study revealed 12 good organizational practices with 31 associated recommendations.

We have drawn attention in the current study to what are good practices to encourage women high-performance coaches. A key finding revealed actions enacted by senior managers are critical to enable good practices to occur. These NSO senior managers include CEOs, directors of high-performance, and managers of high-performance coaches. It is vital senior managers consider the importance of their decision making and practices if they want to attract, develop, and retain women high-performance coaches. Our study revealed historical barriers can be disrupted by these managers through their genuine focus on good practices. The new knowledge presented from our study moves beyond previous research and adds to the theoretical perspective at the EIM organizational level that embeds institutional work conducted by actors working in NSOs. Specifically, the EIM organizational level can move from an emphasis on barriers to good practices identified by the lived experiences of women high-performance coaches and senior managers.

Practically, it is important for senior managers to initiate collaboration with women high-performance coaches in their sport and challenge workplace culture, one that creates strong working relationships between both parties. Encouragement for these women is not a one “one size fits all” approach, every person requires different needs and the good practices identified in our study can be tailored accordingly. It is hoped future studies stimulated by findings from this research, will challenge embedded historical social and cultural beliefs to drive new dialogue and critically analyse NSO practices. Our sporting landscape has the ability to attract, develop, and retain women high-performance coaches, it seems however findings such as those identified in our study need to be clearly translated to assist build knowledge and action by NSOs. The current research can act as a stepping-stone to inculcate strategic organizational practices and assist increase the pace of the number of women high-performance coaches in Australia and more broadly.

## Data Availability

The original contributions presented in the study are included in the article/Supplementary Material, further inquiries can be directed to the corresponding author.
